# Building an Artificial Cardiac Microenvironment: A Focus on the Extracellular Matrix

**DOI:** 10.3389/fcell.2020.559032

**Published:** 2020-09-04

**Authors:** Olivia Pagliarosi, Vittorio Picchio, Isotta Chimenti, Elisa Messina, Roberto Gaetani

**Affiliations:** ^1^Department of Molecular Medicine, Faculty of Pharmacy and Medicine, Sapienza University of Rome, Rome, Italy; ^2^Department of Medical and Surgical Sciences and Biotechnology, Faculty of Pharmacy and Medicine, Sapienza University of Rome, Rome, Italy; ^3^Mediterranea Cardiocentro, Naples, Italy; ^4^Department of Maternal, Infantile, and Urological Sciences, “Umberto I” Hospital, Rome, Italy; ^5^Department of Bioengineering, Sanford Consortium for Regenerative Medicine, University of California, San Diego, San Diego, CA, United States

**Keywords:** cardiac microenvironment, extracellular matrix, cardiac tissue engineering, cardiac differentiation, 3D bioprinting

## Abstract

The increased knowledge in cell signals and stem cell differentiation, together with the development of new technologies, such as 3D bioprinting, has made the generation of artificial tissues more feasible for *in vitro* studies and *in vivo* applications. In the human body, cell fate, function, and survival are determined by the microenvironment, a rich and complex network composed of extracellular matrix (ECM), different cell types, and soluble factors. They all interconnect and communicate, receiving and sending signals, modulating and responding to cues. In the cardiovascular field, the culture of stem cells *in vitro* and their differentiation into cardiac phenotypes is well established, although differentiated cardiomyocytes often lack the functional maturation and structural organization typical of the adult myocardium. The recreation of an artificial microenvironment as similar as possible to the native tissue, though, has been shown to partly overcome these limitations, and can be obtained through the proper combination of ECM molecules, different cell types, bioavailability of growth factors (GFs), as well as appropriate mechanical and geometrical stimuli. This review will focus on the role of the ECM in the regulation of cardiac differentiation, will provide new insights on the role of supporting cells in the generation of 3D artificial tissues, and will also present a selection of the latest approaches to recreate a cardiac microenvironment *in vitro* through 3D bioprinting approaches.

## Introduction

The regulation of cell proliferation, differentiation, and function is influenced by several factors, such as signaling molecules, cell-cell interaction, and the extracellular matrix (ECM), which are collectively defined as the microenvironment. Among their many functions in homeostasis, these factors play a fundamental role also in tissue development, turnover, and repair. The regulation of stem cell behavior is a particularly delicate task: in fact, embryonic stem cells (ESCs) are the main players during embryogenesis, and are tightly regulated and coordinated to maintain their stemness, as well as their activation and functional differentiation. Moreover, the increasing use of induced pluripotent stem cells (iPSCs) to obtain differentiated cell types requires particular attention also on the signals regulating their biology. Indeed, this very specific microenvironment is now commonly defined as the stem cell “niche,” and its understanding and modeling could boost advancements in the field of tissue engineering, and even subsequent *in vivo* therapeutic applications (Chimenti et al., 2017).

The most studied cell regulatory signals are mediated by growth factors (GFs) and soluble cues, widely used in *ex vivo* research to differentiate adult and ESCs into mature cells. The role of molecules such as insulin-like and fibroblast GFs (Bendall et al., 2007), Wnt proteins (Reya et al., 2003; Lie et al., 2005), and many other cytokines (Endele et al., 2014) have been extensively studied, while the role of the ECM in the regulation of the microenvironment and the niche still remains to be fully understood. The ECM is not an inert, structural scaffold, as once thought: it maintains indeed the structure and function of organs, participating in their development and remodeling. ECM allows cell-cell communication and it modulates cell motility, adhesion, survival, and proliferation (Miyamoto et al., 1996; Ross and Borg, 2001). The ECM is composed of structural proteins, such as collagens, and numerous non-structural regulatory proteins, such as proteoglycans (PGs). Within the niche, different ECM molecules can regulate stem cell behavior by direct interactions with integrin receptors expressed on the cellular membrane, by modulating tissue compliance and consequent cell response through the mechanosensing machinery, or through the binding of different GFs and their controlled spatiotemporal presentation to the neighboring cells.

In the heart, ECM proteins surround and protect all cell types: cardiomyocytes, fibroblasts, endothelial cells, pericytes, and smooth muscle cells. All together, proteins, cells, and their cross-talk, create an interconnected microenvironment, which promptly reacts to external changes and cues such as biochemical (GFs, cytokines, hormones), electrical and mechanical signals (such as changes in contraction, stretch, and pressure), necessary to maintain myocardial integrity and functional responsiveness, especially during development and physiological post-natal remodeling (Howard and Baudino, 2013). Cardiac ECM, in fact, is a dynamic structure, constantly undergoing synthesis and degradation, modifying its composition in space and time in response to contingencies, from early development to post-natal life, aging, and possibly diseased states (Schenke-Layland et al., 2012; Rienks et al., 2014).

In this review, we will focus on the role of the ECM in the regulation of the cardiac microenvironment, starting briefly from tissue development during embryogenesis, to the role of different ECM molecules in cardiac differentiation and proliferation, both *in vitro* and *in vivo*. Moreover, we will also address the role of supporting cells and describe the most promising approaches to recreate *in vitro* a physiologically relevant cardiac microenvironment through 3D bioprinting technology.

## The Cardiac Extracellular Matrix

During embryogenesis, the heart is the first functional organ to develop, thanks to the proliferation, migration, and differentiation of cardiac precursor cells (CPCs). These cells migrate from the primitive streak in the embryo, organizing themselves into the mesoderm (Abu-issa and Kirby, 2007), from which the CPCs derive; then they differentiate into all the major cell types of the heart, including cardiomyocytes (CMs), smooth muscle cells, endothelial cells, and fibroblasts (Yustzey and Kirby, 2002; Kelly et al., 2014; Witman and Sahara, 2018). The primitive cardiac tube has an outer layer of myocardium and an inner one of endothelium, divided by an acellular space filled with a provisional ECM, also known as the cardiac jelly, which is essential for the maintenance of the tube structure during its transformation into the definite heart form (Huang et al., 1995). This matrix is enriched in hyaluronic acid (HA) and PGs, which render it highly hydrated and malleable (Bernanke and Markwald, 1984; Funderburg and Markwald, 1986). Cardiac jelly components sequester GFs and influence CPC movement and differentiation, and are extremely important during epithelial-to-mesenchymal transition (EMT), which is necessary for many ontological steps to generate adult tissues and organs (Butcher and Markwald, 2007; Thiery et al., 2009; Votteler et al., 2010). Cardiac jelly also contains fibronectin, laminin, different types of collagen, fibulin, and fibrillin, that are fundamental in orchestrating tissue growth, organization, and differentiation (Icardo and Manasek, 1983; Mjaatvedt et al., 1987; Little et al., 1989; Bouchey et al., 1996).

After birth, the heart undergoes remodeling to adapt to the mechanical, chemical, and electrical changes of the growing body. These alterations in organ geometry are accompanied by changes in ECM composition, and are necessary to maintain efficient cardiac function (Baudino et al., 2006). Many ECM components are involved. Collagen I is the most abundant in both developing and adult tissue. Still, in the latter, its density and crosslinking are higher, and a decrease in the ratio between collagen I and collagen III is observed (Thompson et al., 1979; Borg, 1982; Hanson et al., 2013; González et al., 2019). Fibronectin (FN), instead, decreases in the adult tissue (Kim et al., 1999; Lockhart et al., 2011) with a concomitant increase of elastin (Kim et al., 1999). These changes are generally associated with an increase in cardiac stiffness, with the heart tissue becoming less compliant during heart development and CM differentiation, as demonstrated by the threefold increase of left ventricle stiffness 5 days following birth, up to an elastic modulus of approximately 10–20 kPa (Jacot et al., 2008; Horn, 2015).

Extracellular matrix proteins not only act as anchored ligands: they are also reservoirs for GFs, cytokines, and proteases, in particular matrix metalloproteinases (MMPs) and tissue inhibitor of metalloproteinases (TIMPS) (Hynes, 2009; Brizzi et al., 2012). MMPs degrade collagen (Daley et al., 2008), while simultaneously releasing small bioactive peptides and GFs stored within the matrix, releasing soluble ligands that participate in the homeostasis of the microenvironment. Indeed, FN, vitronectin, collagens, and PGs, avidly bind many GFs, such as FGFs and VEGFs, regulating their presentation, distribution, and activation (Brizzi et al., 2012; Nakayama et al., 2014). Likewise, collagen IV binds BMP4 (Wang et al., 2008).

## Role of the ECM in Cardiac Differentiation

**FIGURE 1:**
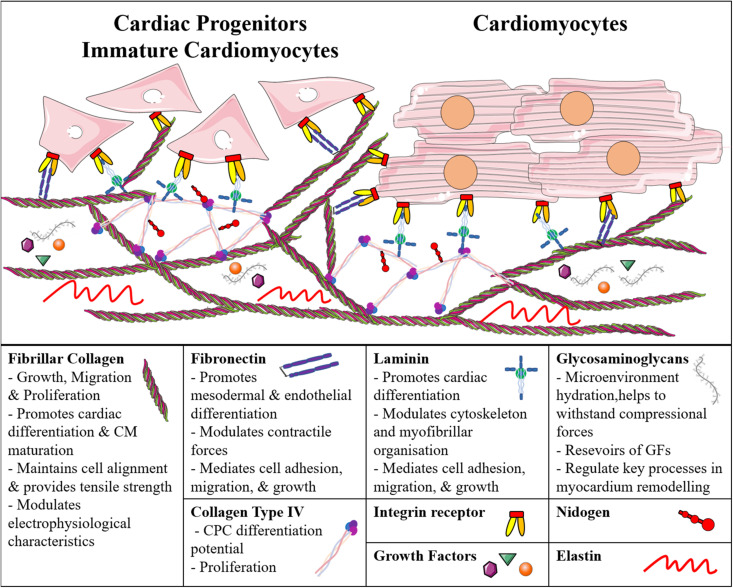
Schematic representation of the main cardiac extracellular matrix components and their direct interaction with immature and adult cardiomyocytes. CM, Cardiomyocyte; CPC, Cardiac Progenitor Cell; GF, Growth Factor.

As mentioned earlier, cells and ECM are directly connected, and the matrix can influence their growth, organization, and differentiation; similarly, the microenvironment regulates stem cell proliferation, hence the maintenance of potency or differentiation into a specific cell lineage (Figure 1).

Cells adhere to ECM proteins through integrin receptors, which activate intracellular signal transductions and are known to regulate many developmentally relevant processes (Wozniak et al., 2004; Saleem et al., 2009; Sa et al., 2014; Neiman et al., 2019). For example, the interaction between collagen type I and β1-integrin was studied in murine iPSC-derived embryoid bodies, and was shown to be important for cell growth and cardiac lineage commitment (Zeng et al., 2013). Collagen type I has been reported to support the maturation of murine (m) and human (h)iPSC-derived CMs, and to modulate intracellular calcium homeostasis and their electrophysiological properties (Lu et al., 2011; Edalat et al., 2019), while collagen type IV can increase the differentiation potential of m/hCPCs (Schenke-Layland et al., 2012). Both of them promote proliferation of CPCs and maintain the pluripotency of mESCs *in vitro* (Lu et al., 2017). It is worth mentioning that even with the same biochemical compositions (e.g., molecular collagen), different microstructures (e.g., fibrous versus porous) can nonetheless differentially affect the phenotype of cardiac cell types (Chimenti et al., 2011).

Similarly, Cheng et al. (2013) described that FN promotes mesodermal differentiation *via* β1-integrin through the activation of the Wnt/β-catenin pathway in mESCs. Laminin (LM), instead, has been described to support ESC-derived CPC differentiation, and modulate the cytoskeleton and myofibrillar organization (Battista et al., 2005; Sa et al., 2014). Similar to single ECM components, culturing ESC-derived CPCs on matrices with different combinations of these ECM proteins has shown efficient cell attachment, cardiac differentiation, and survival (Battista et al., 2005; Jung et al., 2015; Lu et al., 2017). Sa et al. (2014) have investigated the effects of various ratios of FN and LN, and found that human ESCs differentiating on 70:30 FN:LN yielded CMs with higher efficiency compared to simple gelatin coatings. Several commercial ECM-based products are available for *in vitro* studies on substrates with multiple components. Matrigel^TM^, a basement membrane-derived matrix, promotes EMT, generating precardiac mesoderm, and can promote CM differentiation (Zhang et al., 2012). Similarly, Cardiogel^TM^, a mixture of collagen types I and III, LM, FN, and PGs derived from cardiac fibroblasts, leads to better adherence and maturation of CMs (Vanwinkle et al., 1996). Therefore, despite many studies have shown the importance of different ECM molecules in cardiac cell differentiation and functionality, in order to recreate the proper microenvironment *in vitro*, it is essential to provide the cells with all the ECM molecules present in the native tissue. For this reason, tissue-derived biomaterials are increasingly being used to culture both progenitor and differentiated CMs. Different decellularization and recellularization strategies of cardiac tissue or whole heart have been developed, using myocardium originating from rats (Ott et al., 2008; Carvalho et al., 2012; Wang et al., 2019), porcines (Singelyn and Christman, 2010; Ferng et al., 2017; Lee et al., 2017), bovines (Arslan et al., 2018), and even humans (Johnson et al., 2014; Sánchez et al., 2015; Guyette et al., 2016; Masumoto et al., 2016; Tang-Quan et al., 2018). Interestingly, cardiac progenitor stromal cells, isolated from newborn mice or human fetal heart, can increase their proliferative capacity and expression of key cardiac transcription factors when cultured in a cardiac-derived ECM hydrogel compared to collagen only, both in 2D (French et al., 2012) and 3D (Gaetani et al., 2016). Decellularized mouse hearts allow hiPSC-derived CPCs to migrate, proliferate, and differentiate into all cardiac cell types, exhibiting efficient spontaneous beating (Lu et al., 2013). Furthermore, human decellularized cardiac tissue has been shown to support miPSC and mESC viability and differentiation into CMs (Oberwallner et al., 2015). Other authors have exploited slices of decellularized human hearts; for example, Garreta et al. (2016) found that the electrical and mechanical functionality of hiPSC-derived CMs increased when grown on such slices, compared to Matrigel.

Besides biochemical signals, the ECM regulates cell behavior also by modulating the mechanical properties of the microenvironment. Cells respond to mechanical cues by altering their cytoskeletal organization, myofibril striation and alignment, by expressing specific combinations of cell adhesion molecules (CAMs), and activating signaling pathways and mechanosensitive ion channels (Ireland and Simmons, 2015; Tallawi et al., 2015). In fact, CMs differentiate better on substrates of stiffness similar to the native tissue (∼10 kPa) (Engler et al., 2008; Jacot et al., 2008; Bhana et al., 2010; Young and Engler, 2011; Tallawi et al., 2015). Indeed, when the substrate is too rigid or too soft, CMs overstrain themselves or do inefficient work, respectively; in both cases it results in an impaired contractile capacity.

## New Approaches in Modeling the Artificial Microenvironment

As mentioned previously, the regulation of the microenvironment depends on multiple factors such as cell-cell and cell-ECM interaction, as well as GFs, cytokines, and small molecule signaling, that overall modulate cellular phenotypes. Under these premises, many groups have developed novel methods to grow cardiac progenitor and differentiated cells, creating new microenvironments through 3D culture systems, bioprinting, cardiac patches, and organoid chambers.

Among these approaches, 3D bioprinting can generate tissues using different cells or scaffolds simultaneously, thus having the potential to recreate *in vitro*, at least in part, the complex microenvironment of any given tissue through the accurate design of the biochemical properties and structure of biomaterials, together with the proper cellular composition. In the last years, many synthetic and biological materials have been used to make bioinks. Cardiac-derived primitive stromal cells, CPCs, and iPSC-derived CMs have been printed in many types of biomaterials, including alginate, HA, and gelatin, among others, and have been used for regenerative medicine and tissue modeling applications (Gaetani et al., 2012, 2015). As mentioned above, several studies have shown how using a single ECM component fails to recapitulate the complexity of the natural cardiac ECM, and thus cannot efficiently reproduce the innate cellular microenvironment (Gardin et al., 2020). Protocols for decellularized extracellular matrix (dECM) have been recently adapted to be used in conjunction with printing technology. They could represent a very promising scaffold for 3D cell culture, as they retain the complex biochemical composition of the native tissue. In this regard, Pati and Cho (2017) have designed a method to prepare dECM from porcine hearts as a bioink, and have used it to bioprint an MSC-laden construct by extrusion-based 3D printing technology. The resulting dECM had highly preserved levels of collagen and glycosaminoglycans, providing a native-like environment for the maintenance of a 3D structured tissue (Pati and Cho, 2017). The importance of cardiac ECM as a component of bioinks has been also highlighted by Jang et al. (2017), who used cardiac porcine dECM complexed with UV-treated vitamin B2 to prepare cardiac-specific hydrogels. These scaffolds were used in combination with human infant CPCs to fabricate 3D bioprinted cardiac constructs with a stiffness similar to the native myocardium, which enhanced viability, proliferation, and cardiac differentiation of CPCs (Jang et al., 2017). Similarly, Bejleri et al. (2018) have developed a 3D bioprinted patch containing heart dECM for the delivery of human pediatric CPCs. The authors demonstrated that these dECM patches improved cardiogenic differentiation and angiogenic potential when compared to cells grown in gelatin-methacryloyl patches, highlighting the importance of the proper ECM composition in the microenvironment for physiologic cell behavior (Bejleri et al., 2018). Similarly, Yu et al. (2019) described a method to make cardiac dECM bioinks to fabricate patient-specific tissues, with high control over micro-architecture and mechanical properties using a digital light processing (DLP)-based 3D bioprinter. The results showed that dECM bioinks provide a suitable microenvironment to support maturation of hiPSC-derived CMs (Yu et al., 2019).

**FIGURE 2:**
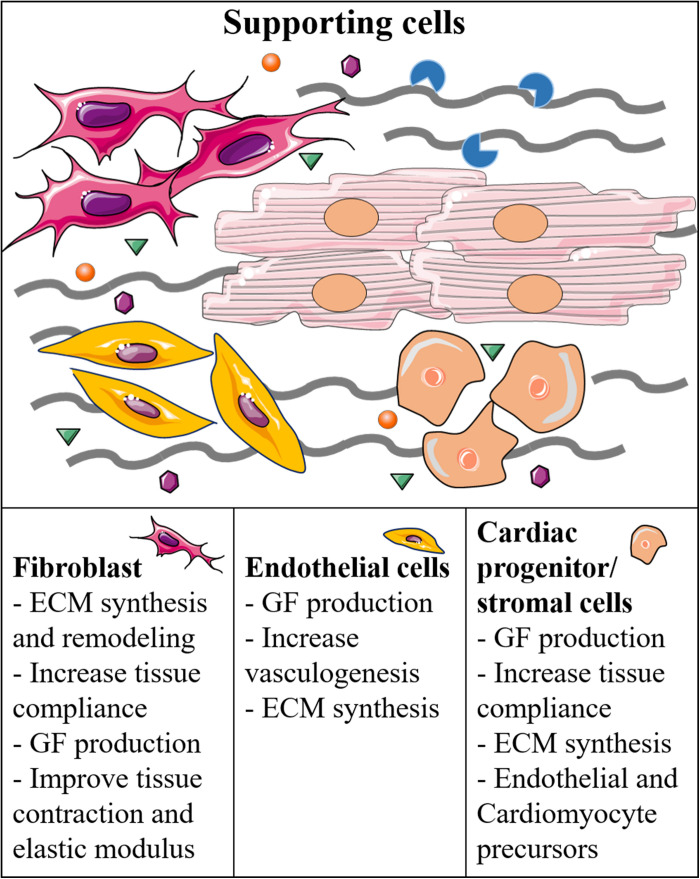
Supporting cells and their function in the regulation of the cardiac microenvironment and generation of cardiac-like tissues.

As mentioned previously, the microenvironment is also modulated by cellular diversity, including supporting cells, such as stromal primitive cells, pericytes, or fibroblasts (Figure 2; Castaldo and Chimenti, 2018; Forte et al., 2018). Several studies in 3D culture systems have shown the beneficial effects of fibroblasts co-culture on ESC- or iPSC-derived CM differentiation (Liau et al., 2011; Ou et al., 2011; Valls-Margarit et al., 2019). These beneficial effects are probably due to the secretion of GFs and deposition of new ECM, which in turn may increase the compaction of the artificial tissues, resulting in better cell-to-cell contact. Furthermore, these systems most likely allow more complex tissue mechanical properties, thereby enabling adequate mechanosensing signaling, which is a fundamental player for tissue maturation (Pesce et al., 2017). Interestingly, Zhang et al. (2019) recently showed that co-culture of hiPSC-cardiac fibroblasts with hiPSC-CMs is beneficial for the electrophysiological properties of CMs compared to co-culture with dermal fibroblasts. The study highlighted the importance of tissue-specificity for boundary conditions in the microenvironment, and could be used not only to improve the maturation of cultured cells, but also to study potential specific mechanosensing signaling mediated by tissue specific cells (Zhang et al., 2019). Besides tissue-specificity, it is also important to consider phenotypic changes due to pathological conditions that may alter the behavior of resident fibroblasts and other stromal cells, and consequently the balance between cardiogenic and fibrotic signaling inside the microenvironment (Pagano et al., 2017). Similar to fibroblasts, also endothelial and smooth muscle cells have been shown to increase the structural or electrical maturation of hiPSC-derived CMs (Giacomelli et al., 2017; Pasquier et al., 2017; Vuorenpää et al., 2017). In fact, hiPSC-derived smooth muscle cells and pericytes could increase the structural maturation of iPSC-derived CMs in a 3D collagen I-matrigel scaffold (Masumoto et al., 2016). Recently, the group has reported a new personalized strategy to engineer immuno-compatible patient-specific cardiac patches. In detail, a biopsy of adipose tissue was isolated from patients, and the cellular and acellular components were separated. While the cells were reprogrammed to become pluripotent stem cells, the ECM was processed into a personalized hydrogel able to support hiPSC differentiation into cardiac cells *in vitro* (Edri et al., 2019). Afterward, the same groups made a more complex 3D model adding endothelial cells as a source of vascular structures, thus obtaining patient-specific vascularized cardiac patches (Noor et al., 2019).

Other examples to generate highly complex structures include the work of Li et al. (2018) that have created for the first time a human ventricle-like cardiac organoid chamber (hvCOC) to generate advanced models resembling cardiac geometry. HiPSC-derived CMs and dermal fibroblasts have been embedded in a collagen-based ECM, and transferred to an agarose mold with a centrally placed inflatable balloon. This allowed to form the hollow 3D hvCOC that enhanced the maturation of ventricular CMs, providing a unique microenvironment that could mimic fluid pumping, similar to a mature heart chamber (Li et al., 2018). Similarly, Macqueen’s team created a tissue-engineered scale model of the human left ventricle with rat ventricle myocytes or hiPSC-derived CMs seeded on a FN-coated scaffold. The scaffold was made by pull spinning a mixture of PCL and gelatin onto a mandrel in the shape of an ellipsoidal ventricle to promote native-like anisotropic myocardial tissue genesis and chamber-level contractile function (Macqueen et al., 2018).

## Conclusion

Reconstructing and mimicking the complexity of native tissues is a challenging objective of contemporary research. Biochemical composition, intercellular communication, co-culture of multiple cell types, and mechanosensing represent key features, among many others, that need to be finely tuned in order to create physiologically relevant microenvironments for cell integration and differentiation. All these features can significantly affect the desired yield and the expected phenotype of artificial tissues and constructs. Increasing knowledge of the basic mechanisms regulating tissue homeostasis, together with advancements in biomaterial preparation and bioprinting, are gradually allowing the design and development of artificial microenvironments with in creasing complexity. This progress will provide innovative tools for *ex vivo* research, as well as new strategies for tissue repair in regenerative medicine.

## Author Contributions

RG, OP, and VP contributed to the text and figures. EM and IC contributed to the text and figure editing. All authors contributed to the article and approved the submitted version.

## Conflict of Interest

The authors declare that the research was conducted in the absence of any commercial or financial relationships that could be construed as a potential conflict of interest.
